# Children's (Mis)understanding of the Balance Beam (Online Edition)

**DOI:** 10.3389/fpsyg.2021.702524

**Published:** 2021-08-23

**Authors:** Virginie M. L. Filion, Sylvain Sirois

**Affiliations:** Groupe de recherche sur la cognition, les neurosciences, l'affect et le comportement (CogNAC) and Département de Psychologie, Université du Québec à Trois-Rivières, Trois-Rivières, QC, Canada

**Keywords:** cognitive development, children, balance-scale task, online testing, rule-assessment, clinical interview

## Abstract

The balance-scale task, proposed by Inhelder and Piaget, illustrates children understanding of weight-distance relationships. Piaget used the clinical interview method in order to investigate children's reasoning. Over the last five decades, Siegler's Rule-Assessment Approach has been used to explain children reasoning in the balance-scale task according to rules children would use to solve the task. However, this approach does not take into account some key perceptual properties of the task. This study evaluates whether different task demands would alter children's errors. Forty children (twenty children aged 4–5 years and twenty children aged 9–10 years) predicted the movement of both arms of 16 balance-scale problems administered online. Nine 4–5-year-olds produced non-plausible responses whereas none of the 9–10-year-olds provided non-plausible responses. These results seem to indicate a basic misunderstanding of the scale from some younger children, one that eludes traditional measures used with this task.

## Introduction

The balance-scale task (Inhelder and Piaget, 1958) is a logicomathematical problem-solving task. The scale consists of two arms in the form of a unitary beam, centrally attached to a fulcrum. On each arm, there are pegs placed at equally spaced distance from the fulcrum which are used to place unit weights. The child's task is to predict whether the left or the right arm will tilt down, or whether the unitary beam will remain in balance. Children's understanding of the weight-distance relationship with force (i.e., the torque applied to the arms) is examined according to their responses.

There are six typical problems used with the balance-scale task (Siegler, 1976). These problems manipulate the weight-distance relationship in different ways. There are three non-conflict problems (balance, weight, and distance) and three conflict problems (conflict-weight, conflict-distance and conflict-balance). In non-conflict problems, at most one parameter (weight or distance) differs on both arms. For weight problems, the weight values differ but distance is the same on each side of the fulcrum. For distance problems, weights are equal on each side but distances differ. In weight and distance problems, the side with relatively largest value tilts down. For balance problems, the values of weight and distance are identical on each arm and the beam remains stable. In conflict problems, the weight and the distance values differ on each arm of the scale. For conflict-weight problems, the arm with relatively more weight creates relatively more torque and tilts down. In conflict-distance problems, it is the arm with the relatively larger distance from the fulcrum that tilts down. Finally, in conflict-balance problems, the combination of weight and distance on each arm creates the same torque and the beam remains stable (Siegler, 1976; Halford et al., 2002).

In line with the general idea of sequential stages of development (Piaget, 2002), it was initially believed that children go through three stages of development in order to solve the task (Inhelder and Piaget, 1958). It was argued that around 5–8 years of age, children acquire an understanding that their actions can impact those of an object. Children thus begin to understand the impact of weight and distance on the scale. However, 5–8-year-olds do not seem to be able to successfully combine the values of weight and distance together. This coordination of information would be understood around adolescence (Inhelder and Piaget, 1958).

The mathematical solution to solve the balance-scale task is to calculate torque. The torque, product of weight and distance, represents the force applied to one side of the scale (Inhelder and Piaget, 1958; Ferretti and Butterfield, 1992; Shultz et al., 1994). The arm with the largest torque will be the one that tilts down. When torques are equal, the beam remains balanced.

Siegler (2016) suggested that development is more like an overlapping wave model. A child could have different strategies (with variable probabilities of use) available at any given time (Siegler, 2016). Siegler and Chen (1998) explain development as a continuum where children dynamically add and select increasingly complex rules. The Rule-Assessment Approach (Siegler and Richards, 1979; Siegler and Chen, 2002) explains that children solve the balance-scale task according to four rules depending on their understanding of the weight-distance relation. Children who have no understanding of the weight-distance relationship would solve the task by chance (Rule 0). They would have an average success rate of 33% on any given trial since they have three answer choices available (i.e., balance, left, or right; Siegler, 1976). Over development, children begin paying attention to weight (Rule 1), then later consider distance when weights are equal (Rule 2), then try and fail to integrate both dimensions (Rule 3), until they successfully compare torques (Rule 4; Siegler, 1976).

Over the years, Siegler's work has been criticized because those four rules explain the reasoning of only 88% of children (Zimmerman, 1999). Jansen and van der Maas (2002) suggested that children can used multiple other rules. There are additive rules, multiplication rules, and perceptual rules (Ferretti and Butterfield, 1986; Jansen and van der Maas, 1997, 2002; Richardson et al., 2006; Messer et al., 2008; Hofman et al., 2015).

The balance-scale task can be an intuitive task if children rely on perception to solve the task (Shultz and Takane, 2007). The torque effect is a perceptual effect caused by the relative salience created on each side of the scale. A bigger difference between the torque of each side of the scale makes it easier for the child to solve the problem (Ferretti and Butterfield, 1986; Hofman et al., 2015).

Task demands could also have an impact on performance (Messer et al., 2008; Hofman et al., 2015). One study examined 4–5-year-olds' basic understanding of the task (Sirois et al., 2005). Using computer-generated images and videos of a balance-scale task where only weight was manipulated (distance was constant on all problems), the authors found that children did not understand the unitary nature of the beam in the apparatus, and given the opportunity would predict impossible behavior from the balance (e.g., both arms down). Published studies use methods that only invite plausible answer choices (Siegler, 1976; Ferretti and Butterfield, 1986; Halford et al., 2002; Hofman et al., 2015). Indeed, recent studies used artificial neural network models (Zon and Xie, 2014; Shultz, 2017; Al-Atrash et al., 2020) to replicate findings of rule-assessment. Children's basic understanding assumption (i.e., the unified character of the scale) remains unchallenged. There is a real possibility that the bulk of the literature on this task has either overestimated children's performance, and/or mischaracterized their errors.

The main objective of this study is to evaluate whether different task demands would reveal different errors. Specifically, we predict that younger children (aged 4–5) do not understand the unified character of the scale (Sirois et al., 2005). Therefore, a proportion of errors will stem from predicting impossible behavior of the scale.

With a different methodology, it is unclear whether the torque effect would remain beneficial, or further compound the misunderstanding of the scale for younger children. Therefore, we manipulate the relative torque across problems, but only predict a beneficial effect for older children.

Finally, for exploratory purposes, we introduced a salient feature to help children focus on the dynamic aspects of the balance-scale, and not just static states. A bell was randomly placed above or below the scale for each child, to create a shift of focus from end states (L, R, or balance) to transformations (upward or downward motion). We predict that this salient feature will affect the types of impossible answers of younger children, given their purported relatively simpler understanding of the scale, if they are nevertheless sensitive to transformations (Sirois et al., 2005). A bell below is expected to enhance their implicit use of torque, whereas a bell above should disrupt it. In both cases, it may provided a finer-grained interpretation of their understanding of the scale.

## Method

### Participants

Forty children participated in the experiment: twenty 4–5-year-olds (13 girls and 7 boys; mean age = 61.1 months, SD = 7.49) and twenty 9–10-year-olds (8 girls and 12 boys; mean age = 116.9 months, SD = 6.09). No child had a diagnosis of learning or developmental disability, and all had normal eyesight and hearing. Children were recruited through Facebook pages that reach parents in various cities of Québec, Canada. All parents had to provide written consent for their child to participate in the experiment. This experiment was approved by the Comité d'éthique en recherche avec des êtres humains de l'Université du Québec à Trois-Rivières.

### Materials and Stimuli

#### Scale

**Figure 1:**
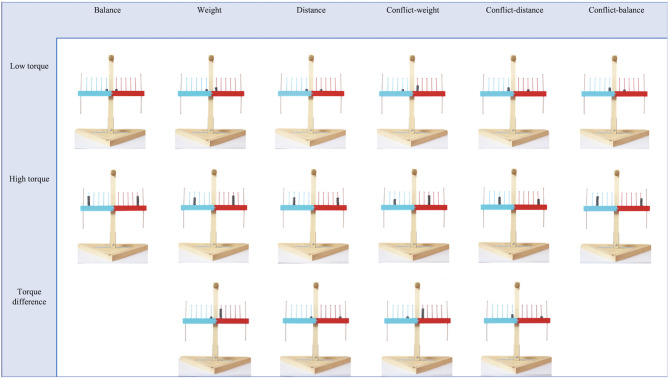
Balance-scale task problems.

A wooden (Figure 1), purpose-built scale 27-inches high and 20-inches wide was used. Each arm of the balance was 10-inches and had six pegs on the top and one on the bottom. Pegs were 1.5-inches apart. The right side and its first five pegs from the fulcrum were red. The left side and its first five pegs were blue. Red and blue pegs were 4-inch high. They were used to place the weights. The four pegs furthest from the fulcrum were brown and 5.5-inches high. They were used to ring a golden bell placed above or below the scale. This bell was at a distance of 11-inches from the fulcrum. As the arms of the scale are one united piece of wood, any difference in torque between left and right arms would cause a single, unified motion (left-down/right-up or left-up/right-down). The weights were hexagonal metal nuts, 0.8-inch in circumference, 0.5-inch high and painted black. These nuts weighed 18 grams. A maximum of five nuts could be put on each peg.

#### Stimuli

Thirty pictures of the scale with different weight-distance configurations on each arm were taken on a white background. There were 16 different types of problems (see examples in Figure 1), and each problem where the weight-distance configuration was different for both arms was duplicated to counterbalance left and right combinations. For problems with alternative images, one of the images was randomly selected for each child. Three types of torque were used in this experiment. The low torque (LT) problems had small weight and distance values on each side of the scale. One trial of each problem types (weight, distance, balance, conflict-weight, conflict-distance, and conflict-balance) was presented. The torque difference (TD) problems had one side with small weight and distance values, the other with large weight and/or distance, creating a large difference between both sides. Problem types of weight, distance, conflict-weight and conflict-distance were used with TD (no balance problems can be created for TD trials). Finally, the high torque (HT) problems had large weight and distance values on each arm. Torque differences were also larger for the HT problems than for corresponding LT problems. Each of the six problem types were presented in the HT subset. The order of the 16 problems presented to the children was counterbalanced and randomized. Bell position was randomized between children.

### Procedure

The experiment was a 15-min meeting on the videoconferencing platform Zoom. A script was developed to standardize the procedure across participants. The experiment began with a presentation of the scale. The researcher showed the position of the bell to the child. A short demonstration allowed the child to hear the sound of the bell when the scale tilted either side. Then, in the manipulation phase, the child could see five simple movements of the scale. Each time children saw the movement, they had to explain what the balance did. After the demonstration, the child was invited to choose three weight-distance configurations of their choosing and test the scale behavior for each.

Then, 16 pictures of the scale were sequentially presented to the child, who had to predict the movement for each side of the scale. For each picture, the child was asked “what does the blue side do” and “what does the red side do.” The question order (red then blue, or blue than red) was counterbalanced and randomized for each child.

To keep children engaged, there were seven predetermined encouragements during the experiment (e.g., “good,” “you are doing fine”). Times of encouragements (after trials 1, 4, 8, 10, 12, 14, and 16) were chosen randomly. They were independent of performance, so should not introduce systematic biases.

Children could take breaks if needed or stop the experiment at any time. Both age groups had the same procedure. Children were allowed to change their answers when they considered they made a mistake on their first attempt. Their second answer was, then, used for the analysis. At the end of the experiment, the child and parent were thanked by the researcher.

### Data Preprocessing

Raw data were compiled using Matlab. Performance on each trial was scored 1 when correct, 0 otherwise. Non-plausible answers are erroneous responses whereby children predicted a violation of the rigid and unitary nature of the arm. They were coded as “BothDown,” “LeftDown,” “RightDown,” “BothUp,” “LeftUp,” and “RightUp.” Responses coded as “BothDown” involve a prediction of both arms down. The code “LeftDown” means the child predicted the left arm went down and the right arm remained stable. For “LeftUp,” the child would have predicted that the left arm went up and the right remained stable. All implausible errors involving downward motion were tallied into “TotalDown” scores; those related to upward motion were tallied into “TotalUp” scores.

Children were also classified according to Siegler's rules. Rules 0–4 create unique sets of predictions (correct, wrong, guess) for each of the 16 problems. Using Euclidean distance (e.g., Aldenderfer and Blashfield, 1984), the square average distance between children's performance on all 16 trials (1 for correct, 0 for wrong) and the predictions from each rule for those trials (1, 0, and 0.333 respectively for correct, wrong, and guess) were computed. Children were assigned the rule associated with the least Euclidean distance from their performance (see Supplementary File for details).

## Results

Out of 16 problems, the mean number of correct answers was 8.5 (95%CI [7.42; 9.57]). Younger children (4–5-y-o) had an average of 6.05 correct answers (95%CI [4.93; 7.17]). Older children (9–10-y-o) had an average of 10.95 correct answers (95%CI [9.91; 11.99]). An independent-sample *t*-test revealed a significant difference between the two age groups, *t*(38) = −6.72, *p* < 0.001, Cohen's *d* = −2.13.

Table 1 shows children classification according to Siegler's rules. A Chi-square test of independence indicated a significant association between Siegler's rules and children's age [*X*^2^(4) = 17.28 *p* = 0.002, *V* = 0.66].

**Table 1:** Observed and expected children's classification according to Siegler rules.

**Groups**	**Effectives**	**Siegler rules**				
		**0**	**1**	**2**	**3**	**4**
4–5-year-olds	Observed	12	6	1	1	0
	Expected	7.0	5.0	5.0	1.5	1.5
9–10-year-olds	Observed	2	4	9	2	3
	Expected	7.0	5.0	5.0	1.5	1.5
Total	Observed	14	10	10	3	3
	Expected	14.0	10.0	10.0	3.0	3.0

Younger children (aged 4–5) produced 41 non-plausible responses whereas 9–10-year-olds did not provide non-plausible responses. A Chi-square test of independence indicated a significant association between the group age and the production of non-plausible responses [*X*^2^(1) = 11.61 *p* < 0.001, *V* = 0.54]. According to a Chi-square goodness of fit test, there was a significant number of 4–5-year-olds children who produced non-plausible responses (*N* =9) [*X*^2^(1) = 4 036.06 *p* < 0.001]. A Friedman analysis found no significant difference between the types of torque in non-plausible responses among 4–5-year-olds [*X*^2^(2) = 1.23, *p* = 0.54].

**Figure 2:**
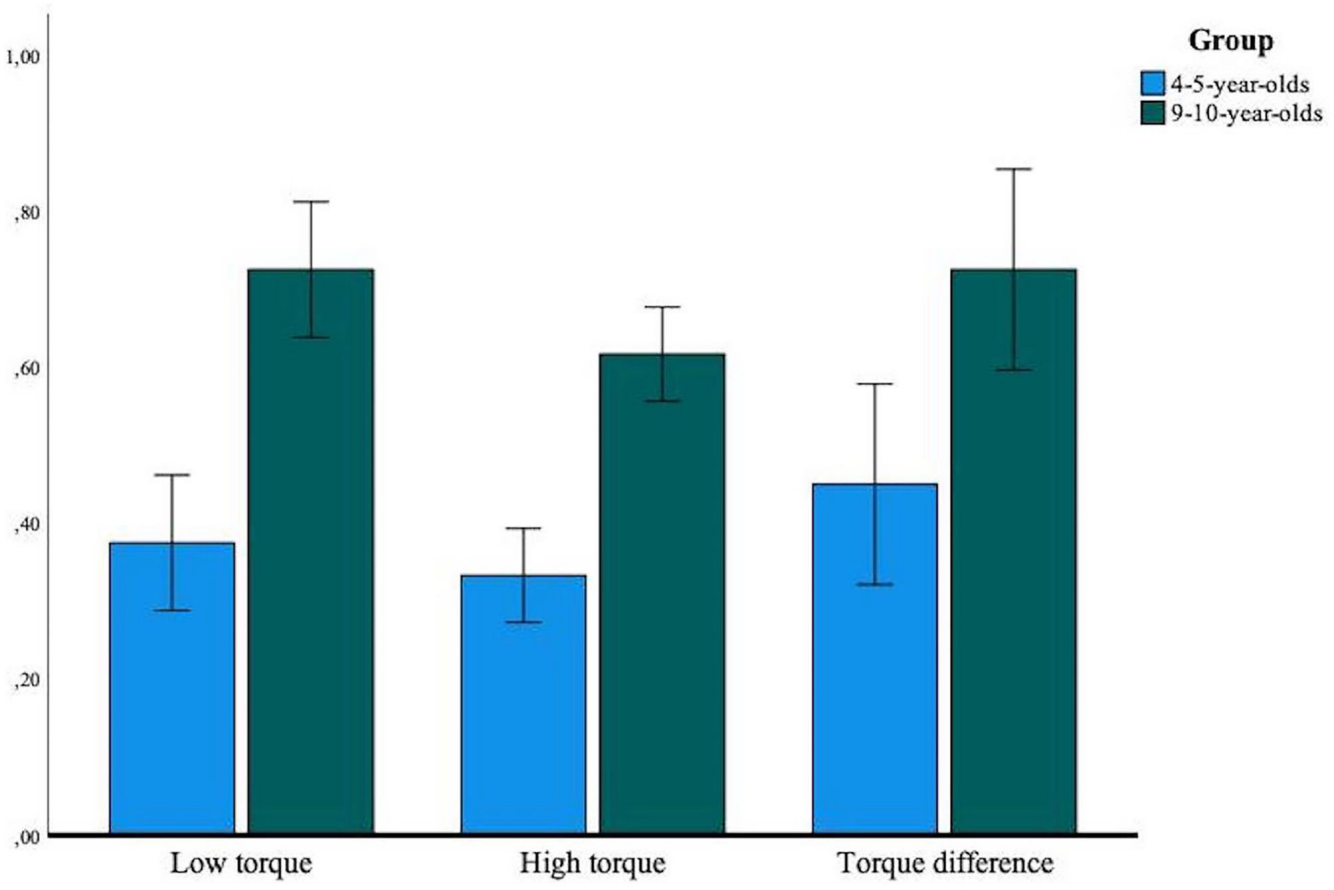
Mean group success rate by torque type. Error bars represent the 95% confidence intervals.

Figure 2 presents the mean success rate by torque type for both age groups. A mixed ANOVA indicated no significant interaction between types of torque and group, [*F*_(2, 76)_ = 0.52, *p* = 0.56, η^2^ = 0.01]. Planned contrasts were used to assess the differences between the types of torque. LT (*M* = 0.55, *SD* = 0.19) success rate was not significatively different than TD (*M* = 0.59, *SD* = 0.19) success rate, [*F*_(1, 38)_ = 0.74, *p* = 0.40, η^2^ = 0.02]. However, LT success rate differed significatively from HT (*M* = 0.48, *SD* = 0.19), [*F*_(1, 38)_ = 7.28, *p* < 0.01, η^2^ = 0.16].

**Figure 3:**
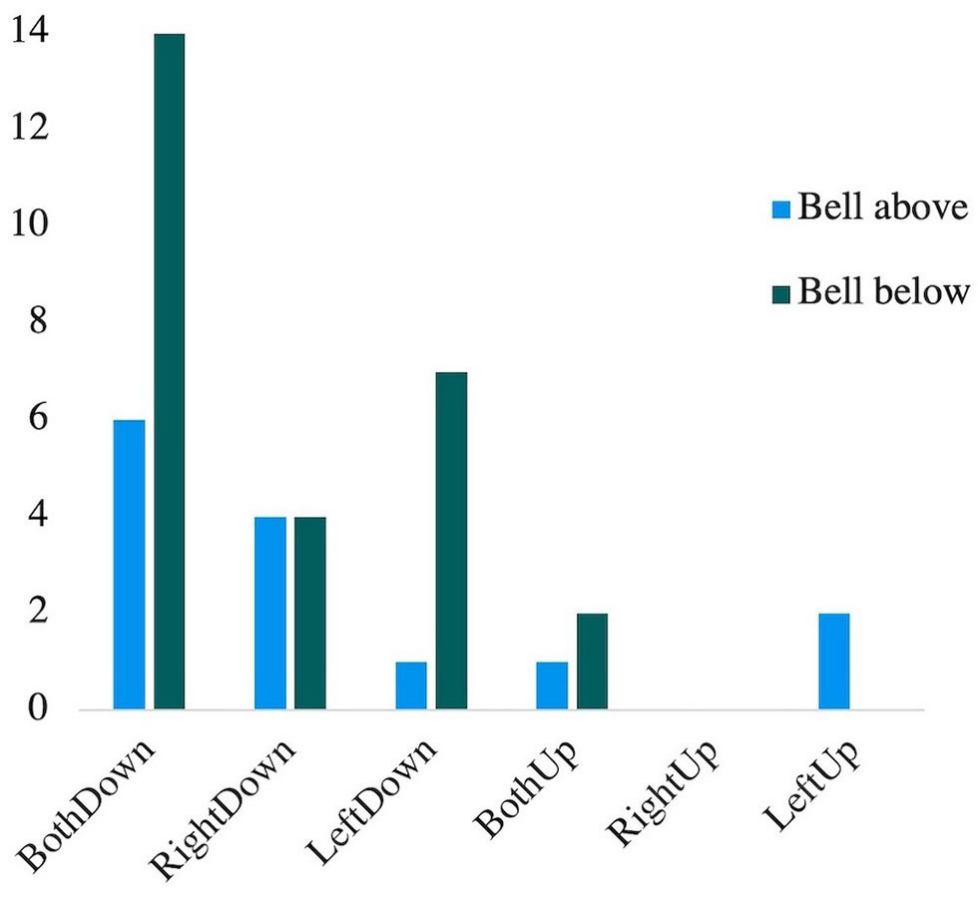
Number of implausible answers by bell position.

Figure 3 shows the 41 non-plausible responses of the younger children as a function of the position of the bell. The association between non-plausible answers and the position the bell was tested with a Chi-square goodness of fit test. “TotalDown” responses (*N* = 36), differed significantly by bell position [*X*^2^(1) = 5.44 *p* < 0.05, *V* = 0.39], but not “TotalUp” (*N* = 5) [*X*^2^(1) = 0.2 *p* = 0.66, *V* = 0.2].

## Discussion

The present study is consistent with previous findings, as most 4–5-year-olds seemed align with Rules 0 or 1, and 9–10-year-olds with Rules 1 or 2. The impact of weight on the scale is easily understood by children because it is more salient and relevant in their environment and experiences (Inhelder and Piaget, 1958; Siegler, 1976; Ferretti and Butterfield, 1986; Halford et al., 2002; Jansen and van der Maas, 2002). The contribution of distance takes more time to notice and integrate (Leuchter and Naber, 2019). Thus, it is possible to observe Rule 1 until the age of 11 (Siegler and Chen, 1998; Jansen and van der Maas, 2002; Leuchter and Naber, 2019).

Three children in this study were classified with Rule 4. Before age 14, children do not typically understand the torque rule (Siegler and Chen, 1998; Jansen and van der Maas, 2002). Children who resolved the conflict problems could have succeeded by intuition (Messer et al., 2008; Dandurand and Shultz, 2009; Hofman et al., 2015). Children seem to be able to solve the problems without being able to verbalize their reasoning (Messer et al., 2008). Ironically, this is a departure from Piaget's clinical interview method, whereby reasoning is assessed by explanation (Posner and Gertzog, 1982).

The rule classification results we report are important in two respects. First, we deviate from standard approaches used with the balance-scale task by asking children to process each arm independently. Despite this departure, the differences between age groups in our study mirror findings from traditional rule-assessment methods. Arguably, our task measures the same cognitive abilities. Those results suggest that the present method is an adequate version of the balance-scale task. Second, we used an online testing approach to accommodate in-person testing restrictions during the Covid-19 pandemic. Unlike children tested in person, our sample were not able to physically manipulate the balance scale themselves (although they were given three trials to ask the experimenter to manipulate it for them as they saw fit). Rule classification results suggest that online testing can assess similar abilities to what is normally measured in the lab. This is particularly relevant for inclusivity imperatives, whereby online testing can help solve the so-called WEIRD problem in psychology (Jones, 2010). The balance-scale task could be used online to reach typically underrepresented groups. Unfortunately, the minimal demographic information collected in this preliminary study does not allow to assess inclusion. Further work, with appropriate recruitment strategies, is required to assess inclusiveness targets.

As noted, Siegler's rules approach does not explain some kinds of errors children can produce in the balance-scale task (Sirois et al., 2005; Boom and ter Laak, 2007). In standard studies, children's understanding of the scale and their reasoning is based on the prediction of the movement of the scale using a restricted set of plausible (albeit not necessarily correct) answer choices. Children are presented three possible answers (i.e., right arm goes down, left arm goes down or both arms stay stable). However, in the present study, children had to predict the movement of each arm. They could process one arm independently of the other arm. This methodology can lead to a more detailed understanding of the child's reasoning.

We predicted that younger children who do not understand the unified character of the scale would suggest non-plausible behavior of the scale (an interesting type of error, insofar as characterizing their thinking, that is not allowed in standard task protocols). The older children were not expected to make those errors. The non-plausible responses produced by younger children seem to confirm the hypothesis. There were 9 out of 20 of 4–5-year-olds who provided responses that are implausible. About half of younger children seemed to process each arm independently of the other arm. The misunderstanding of the unified character of the scale could be caused by the focus of children on their own action. Children aged 3–5 conflate their own actions with those of other objects (Piaget, 1928; Inhelder and Piaget, 1958). In our task, the action of children is divided in two answers, so it could mean that, for them, the balance has also two different actions.

When children misunderstand the unified character of the scale, it appeared associated with a salient feature (i.e., a bell placed above or below the scale). Most non-plausible responses were due to children predicting both arms going down, and were primarily associated with the bell located below the scale. It seems that children understand gravity due to their daily life experiences of the downward pull of weight (Halford et al., 2002). Salient features can lead younger children to focus on a specific aspect of the task (Piaget, 1928, 2002; Amsel et al., 1996). If there was a focus on the transformation (i.e., ring the bell), in relation to their knowledge, this could explain part of the presence of non-plausible responses. It would be interesting to verify this exploratory finding in future studies that use upward force to manipulate a balance-scale, and whether this would be associated with more upward non-plausible responses. At this time, a cautious conclusion is that an incidental salient feature can affect performance on the balance-scale, which could be uniquely useful for a finer-grained analysis of children's understanding.

In previous studies, the saliency caused by the bigger torque on one arm seemed to facilitate the choice between the three possible answers for children (i.e, the torque effect; Ferretti and Butterfield, 1986; Jansen and van der Maas, 2002; Shultz and Takane, 2007; Li et al., 2017). When children's task is to predict the movement of both arms, we expected that the torque effect would not occur for younger children who misunderstand the scale, but that it would be present for older children. Results suggest that the torque effect fades when children of all ages have to process information from both arms to predict their movement. This effect could be explained by different process for both age group. The encoding ability for younger children is less efficient (Boom and ter Laak, 2007). When multiple stimuli are presented, they do not seem able to encode all information at the same time (Siegler, 1976; Amsel et al., 1996). In standard studies, children can choose which arm to process and ignore information from the other arm. However, when younger children are required to specifically process one arm, they will only take into consideration information from this arm. After, they will process the other arm independently of the first one they processed. Therefore, children could miss the salience of the difference between the two torques because they do not have a global perspective allowing for relative comparisons.

Thus, there is no evidence of a torque effect in the present study. For older children, the success rate for torque difference and low torque were similar. Older children can more easily process information of both arms at the same time (Amsel et al., 1996), but the present task imposed a stepwise reasoning approach. Sometimes, older children gave an answer for one arm and, when they had to give an answer for the second arm, they would change their first answer to ensure a better fit. It happened for most 9–10-year-olds, but it was not documented. It would be useful in future work to include that metric to understand when and how many times children use that strategy. It is possible that a bigger difference between the two arms still facilitated responses, but that low torque is also facilitated because of the methodology. The possibility to take time to process both arms could have increased the success rate of low torque as well.

The high torque trials seem to have a lower success rate than the other types of torque. It could be explained by the perceptual properties of that torque. Both arms are saliant in that type of torque. The force applied to the scale on both arms could increase the difficulty of the problems for children of both ages. Children could have made an association between large torque and downward motion. However, with two high-torque arms, it would be relatively difficult to understand the problem, leading to errors.

The interpretation of our findings must be done with caution. The thinking of children seems variable between and within studies according to methodological differences (Halford et al., 2002; Messer et al., 2008; Bullard, 2009; Zon and Xie, 2014). Asking to predict the movement of both arms could explain our results, but it needs to be replicated to assess when children understand the unified nature and behavior of the scale. In a future experiment, it would be useful to add a practice phase after the manipulation phase, as children need time to properly understand a task (Jansen and van der Maas, 2002).

The present study adds information about the nature of children's thinking when the balance-scale task is altered. Perceptual properties of the task do affect children's performance (Halford et al., 2002; Messer et al., 2008). Children base their answers on their intuition, which is substantially about the visual properties of the presented problem (Bullard, 2009). However, those perceptual properties can lead children to errors in their reasoning. Importantly, decades of research with this task may have overestimated the competence of younger children, as the task demands of standard studies minimize potential errors that have uniquely been revealed in the current study.

## Data Availability Statement

The raw data supporting the conclusions of this article will be made available by the authors, without undue reservation.

## Ethics Statement

The studies involving human participants were reviewed and approved by Comité d'éthique en recherche avec des êtres humains de l'Université du Québec à Trois-Rivières. Written informed consent to participate in this study was provided by the participants' legal guardian/next of kin.

## Author Contributions

VF and SS designed the study and performed the statistical analysis and wrote the manuscript. VF prepared the stimuli, recruited participants, and collected the data. SS wrote the Matlab scripts to process and compile data. All authors contributed to the article and approved the submitted version.

## Conflict of Interest

The authors declare that the research was conducted in the absence of any commercial or financial relationships that could be construed as a potential conflict of interest.

## Publisher's Note

All claims expressed in this article are solely those of the authors and do not necessarily represent those of their affiliated organizations, or those of the publisher, the editors and the reviewers. Any product that may be evaluated in this article, or claim that may be made by its manufacturer, is not guaranteed or endorsed by the publisher.
