# Supplementary material for: Children's (Mis)understanding of the Balance Beam (Online Edition)
Source: Front Psychol. 2021 Aug 23;12:702524. doi: 10.3389/fpsyg.2021.702524 (PMC8419348; doi:10.3389/fpsyg.2021.702524)
Supplement: Supplementary file 1 [file Data_Sheet_1.pdf]

Rule assessment procedure

Children's performance on each of the 16 trials can be scored as correct (1) or incorrect (0) based on their predictions for both arms of the balance scale. In order to assess which of the Siegler rules best describes the performance of an individual child, we can use the Euclidean distance between the performance of the child and the predicted performance of each rule (Aldenderfer & Blashfield, 1984). The rule with the least distance from the child's performance is considered most similar, and assigned to that child.

Let R_0, R_1, …, R_4 be the five prediction vectors based on Rules 0 to 4. Each vector has 16 entries, corresponding to the prediction of the rule for each problem. Thus, for Rule 0, the prediction vector takes the form

$R_0 = [R_{0,1}\ R_{0,2}\ \dots\ R_{0,16}]$

where $R_{0,1}$ is the prediction of Rule 0 for problem 1, and so forth. Each of the five prediction vectors has the same structure.

Each entry in the prediction vectors is either 1 when the performance should be correct, 0 when it should be wrong, or 0.333 when the child is expected to guess. When children guess, they have 3 options (tip left, tip right, and balance). Thus 1/3 = 0.333.

Rule 0 predicts that children guess on all trials, and so the prediction vector has the following entries

$R_0 = [0.333\ 0.333\ \dots\ 0.333]$

whereas Rule 4 for example predicts that children succeed on all trials and has the following entries

$R_4 = [1\ 1\ \dots\ 1]$

We have constructed the five prediction vectors from the predictions of Rules 0-4 that apply to each of the 16 problems. Similarly, the performance of a given child on the task can be expressed as the vector

$X = [x_1\ x_2\ \dots\ x_{16}]$

where x_1 is his or her performance on problem 1, and so forth.

This allows us to compute the similarity of a child's performance with each of the rules with the following Euclidean distance equation[1]

$$S_j = \frac{\sum_{i=1}^{16} \left(x_i - R_{j,i}\right)^2}{16}$$

where S_j is the similarity of the child's performance to Rule j, expressed as the average squared distance of the performance from the prediction, x_i is the performance of the child on the ith problem, and $R_{j,i}$ is the prediction of Rule j on problem i. The rule with the smallest S_j value is the one that most resembles the performance of the child.

---

[1] We prefer the average squared distance for arbitrary reasons. The more common procedure of taking the square root of the sum of squared distances produces the same relative ordering of distances from each rule. Since we are dealing with squares (and thus positive numbers), if $a^2 > b^2$, then it follows that $a > b$. Likewise, in that scenario, $a^2/c > b^2/c$. Therefore, how to compare the relative sizes of a and b can be a matter of personal preference.
